# Therapeutic Administration of *Malva parviflora* Hydroalcoholic Extract Restores Cognitive Function Consistent with a Resilient Phenotype in Female 5XFAD

**DOI:** 10.3390/ijms27198665

**Published:** 2026-09-28

**Authors:** Itzel Rojas-Sánchez, Eladio Cortes-Flores, Nohemi Camacho-Concha, Jorge Luis Almazán, Leticia Ramírez-Lugo, Esther Crivelli-Galindo, Cristina E. Ramirez-Serrano, Gustavo Pedraza-Alva, Leonor Pérez-Martínez

**Affiliations:** 1 Laboratorio de Neuroinmunobiología, Departamento de Medicina Molecular y Bioprocesos, Instituto de Biotecnología, Universidad Nacional Autónoma de México, Cuernavaca 62210, Morelos, Mexico; itzel.rojas@ibt.unam.mx (I.R.-S.); eladio.cortes@ibt.unam.mx (E.C.-F.); nohemi.camacho@ibt.unam.mx (N.C.-C.); jorge.ochoa@ibt.unam.mx (J.L.A.); esther_crivelli@ciencias.unam.mx (E.C.-G.); cristina.ramirez@ibt.unam.mx (C.E.R.-S.); gustavo.pedraza@ibt.unam.mx (G.P.-A.); 2 Departamento de Neurociencia Cognitiva, Instituto de Fisiología Celular, Universidad Nacional Autónoma de México, AP 70-253, Ciudad de México 04510, Ciudad de México, Mexico; llugo@ifc.unam.mx

**Keywords:** Alzheimer’s disease, *Malva parviflora*, long-term potentiation, synaptic plasticity, memory, neuroinflammation, resilient phenotype, reactive astrogliosis, therapeutic treatment, female 5XFAD

## Abstract

The remarkable increase in human life expectancy has been accompanied by a rising global incidence of neurodegenerative disorders, particularly Alzheimer’s disease. This condition is characterized by progressive accumulation of soluble and insoluble amyloid-beta peptides, neuroinflammation, and synaptic dysfunction in the hippocampus, leading to impaired higher-order cognition. Epidemiological evidence indicates that women are disproportionately affected; however, female-specific pathology remains understudied. Here, we evaluated the therapeutic potential of a hydroalcoholic extract of *Malva parviflora* in female 5XFAD mice following prolonged intragastric administration initiated after disease establishment. Long-term treatment shifted cognitive performance toward a phenotype comparable to wild-type littermates, an improvement associated with reduced soluble amyloid-beta levels in the hippocampus and peripheral circulation, and significant attenuation of reactive astrogliosis as evidenced by reduced hippocampal glial fibrillary acidic protein levels. Because reactive astrocytes impair synaptic activity and metabolic homeostasis during disease progression, we examined the direct effects of *Malva parviflora* on synaptic plasticity ex vivo. Incubation of hippocampal synaptosomes from adult female 5XFAD mice with the extract restored chemically induced long-term potentiation. These findings demonstrate that *Malva parviflora* attenuates key pathological hallmarks of Alzheimer’s disease and promotes cognitive recovery, supporting its potential as a plant-derived therapeutic candidate, particularly in females most affected by Alzheimer’s disease. Future studies including both sexes under a therapeutic paradigm are needed to determine the generalizability of these findings.

## 1. Introduction

Women account for approximately two-thirds of all Alzheimer’s disease (AD) cases (Alzheimer’s Association). Women diagnosed with AD exhibit faster cognitive decline than men, suggesting greater vulnerability to AD pathology and underscoring the need for targeted therapeutic interventions that account for sex differences in disease presentation [1].

AD is characterized by extracellular deposition of amyloid-β (Aβ) peptides and intracellular accumulation of hyperphosphorylated Tau, which collectively drive astrocyte- and microglia-mediated increases in pro-inflammatory cytokines including IL-1β, IL-18, IL-6, and TNF, establishing a neuroinflammatory process that results in inhibition of long-term potentiation (LTP), reduced neuronal branching, and cognitive impairment [2,3,4,5]. Critically, soluble Aβ species, rather than insoluble fibrillar plaques, are now recognized as the primary drivers of synaptic dysfunction and cognitive impairment in AD, acting through disruption of glutamatergic receptor trafficking, synaptic plasticity, and LTP induction [6,7]. Among the pro-inflammatory mediators implicated in AD pathogenesis, IL-18 warrants particular attention, as it has been shown to promote amyloid precursor protein (APP) processing and Aβ production, establishing a feedforward loop between neuroinflammation and amyloidogenesis [8]. In addition to amyloid pathology and neuroinflammation, cholinergic system dysfunction represents a hallmark of AD that directly contributes to cognitive decline. The loss of cholinergic neurons in the basal forebrain and the associated reduction in acetylcholine signaling in the cortex and hippocampus are among the earliest and most consistent findings in AD and correlate with the severity of cognitive impairment [9]. Strategies that preserve cholinergic neuronal morphology and function may therefore represent a complementary therapeutic target. In this context, glial fibrillary acidic protein (GFAP) positive reactive astrocytes have been proposed to play a central role in orchestrating memory decline, particularly in females; higher GFAP levels have been associated with worse global cognition in women compared with men [10]. Furthermore, Flores et al. [11] demonstrated that despite persistent neuroinflammation and Aβ accumulation, caspase-1 inhibition was sufficient to restore episodic and spatial memory in 12–15-month-old female and male J20 APPSw/Ind mice, an effect accompanied by recovery of dendritic spine density and the synaptic marker synaptophysin, collectively suggesting that rescue of synaptic functionality is sufficient to restore cognitive function in advanced-stage AD, independently of the neuroinflammatory state.

Current AD therapies offer limited efficacy, particularly at late stages of disease when pathology is already well established, highlighting the urgent need for therapeutic strategies capable of attenuating disease progression after symptom onset. Plant-derived natural products with favorable safety profiles have garnered increasing interest as candidates to improve synaptic function and attenuate cognitive impairment in AD. We previously demonstrated that a hydroalcoholic extract of *Malva parviflora* leaves (MpHE), an herbaceous plant rich in flavonoids and phenolic compounds, exerts neuroprotective effects in male 5XFAD mice fed a high-fat diet, reducing astrogliosis and sustaining an anti-inflammatory microglial phenotype. MpHE also reestablished microglial phagocytosis through a PPARγ/CD36-dependent mechanism, resulting in reduced Aβ accumulation and improved hippocampal-dependent spatial memory [12]. Importantly, these effects were demonstrated in a preventive paradigm initiated prior to the establishment of full AD pathology [12].

The 5XFAD mouse model, which carries five familial AD-associated mutations that act additively to accelerate amyloid pathology, is particularly relevant to the present study. Female 5XFAD mice develop early cognitive deficits (4-month-old), making them an appropriate model for investigating therapeutic strategies in the sex most severely affected by this disease [13]. Given that AD disproportionately affects women and that effective therapeutic interventions for late-stage disease remain scarce, here, we extended the investigation of MpHE to a therapeutic paradigm in female 5XFAD mice, which is both scientifically justified and clinically relevant.

In the present study, we demonstrate that therapeutic intragastric administration of MpHE initiated after the establishment of AD pathology improves episodic-like and working memory in female 5XFAD mice. The neuroprotective effect of MpHE on memory performance was associated with reduction in soluble Aβ peptides, attenuation of astrogliosis and IL-18 signaling, promotion of cholinergic neuronal branching, and rescue of hippocampal LTP without modification of Aβ plaque burden or local inflammatory cytokine gene expression, supporting the view that synaptic dysfunction represents the closest functional correlate of cognitive impairment in AD and positioning MpHE as a candidate therapeutic intervention for advanced-stage AD.

## 2. Results

### 2.1. Malva parviflora Hydroalcoholic Extract Treatment Initiated After Disease Establishment Does Not Modify Amyloid Plaque Burden or Hippocampal Inflammatory Gene Expression in 5XFAD Female Mice

We have previously shown that MpHE has anti-inflammatory effects, restoring microglial function via a PPARγ-dependent mechanism, highlighting its preventive potential in male 5XFAD model [12]. However, whether MpHE can exert therapeutic effects once AD pathology is established remains unknown. Because female 5XFAD mice develop pathological and cognitive alterations earlier and more severely than males [13], we focused our investigation on this sex. The 5XFAD model carries five familial AD-associated mutations that act additively to promote excessive Aβ production from approximately 1.5 months of age, Aβ plaque deposition from approximately 2 months, and synaptic loss, memory impairment, and cognitive deficits from approximately 4 months of age. Accordingly, MpHE was administered intragastrically once daily beginning at 6.5 months of age, when cognitive deficits are already well established (Figure 1A; Supplementary Figure S1A).

After 5 months of MpHE treatment, extracellular Aβ plaque burden, a hallmark of AD, remained unchanged in the total hippocampus of 5XFAD female mice compared with vehicle (Vh)-treated controls (Figure 1B,C). Regional analysis of the dorsal subiculum (DS), dentate gyrus (DG), hippocampal CA1, and CA3 similarly revealed no significant differences between MpHE- and Vh-treated 5XFAD female mice (Figure 1D). At the transcriptional level, *il-1β* (*p* = 0.002) and *il-6* (*p* = 0007) mRNA expression was elevated in 5XFAD female mice relative to WT controls; however, MpHE treatment did not modulate the expression of either gene (Figure 1E–G). Collectively, these findings indicate that late-stage MpHE treatment does not modify these key pathological hallmarks of AD.

### 2.2. Malva parviflora Hydroalcoholic Extract Decreases Soluble Amyloid-β Peptide Levels and Attenuates Astrogliosis in 5XFAD Female Mice

The above results highlight the importance of treatment timing and support the notion that the therapeutic benefits of MpHE may involve mechanisms independent of amyloid plaque clearance or local inflammatory cytokine reduction. We therefore evaluated GFAP, an astrocytic cytoskeletal protein proposed as a biomarker of AD [14]. GFAP protein levels were significantly elevated in 5XFAD female mice (*p* ≤ 0.0001), and MpHE treatment reduced GFAP levels by 29.4% in these animals (Figure 2A upper panel, Figure 2B upper panel).

APP was absent in WT mice but highly expressed in 12-month-old 5XFAD female mice. MpHE treatment significantly reduced APP accumulation in 5XFAD mice (*p* = 0.04). Consistent with this, MpHE treatment significantly reduced soluble Aβ40–42 monomer levels in the hippocampus of 5XFAD female mice (*p* = 0.032) (Figure 2A middle and lower panel, B lower panel) and correlated with a reduction in serum Aβ42 levels in MpHE-treated 5XFAD female mice (*p* = 0.022) compared with Vh-treated controls (Figure 2C).

APP accumulation and Aβ production have previously been reported to be promoted by IL-18 [8], a pro-inflammatory cytokine overexpressed in AD patients [15]. We therefore quantified serum IL-18 levels in WT and 5XFAD female mice treated with MpHE or Vh. IL-18 levels were significantly elevated in 5XFAD females compared with WT controls (*p* ≤ 0.05), and MpHE treatment reduced serum IL-18 levels below those of WT females (*p* ≤ 0.005) (Figure 2D). Except IL-6, serum levels of CCL17, CCL22, CXCL1, TGF-β, and IL-12p40 remained unchanged across groups (Supplementary Figure S2A–F).

Taken together, these results indicate that MpHE treatment reduces systemic and hippocampal soluble Aβ peptide levels and attenuates astrogliosis in 5XFAD female mice, a finding that raises the hypothesis that modulation of IL-18 signaling may be a contributing mechanism; however, a causal relationship between MpHE, IL-18 reduction, and soluble Aβ clearance has not been established in the present study and requires direct experimental testing.

### 2.3. Malva parviflora Hydroalcoholic Extract Promotes Cholinergic Neuronal Branching and Restores Long-Term Potentiation at Synaptic Terminals in Advanced-Stage AD

We previously demonstrated that MpHE exerts neuroprotective effects in 5XFAD mice, reducing cognitive impairment and improving learning and memory [12]. Additionally, scopoletin, a bioactive compound identified in MpHE [12], has been reported to directly modulate neurotransmitter release from synaptosomes and enhance LTP [16]. These observations prompted us to investigate whether MpHE could also improve neuronal morphology and synaptic function.

Given the critical role of cholinergic neurons in maintaining cognitive function and regulating cortical signal transduction, we evaluated the effects of MpHE in SN56 cells, a murine cholinergic neuroblastoma cell line derived from septal neurons able to differentiate into a cholinergic phenotype [17]. Based on prior studies demonstrating anti-inflammatory effects of MpHE at concentrations between 100 and 1000 µg/mL without compromising cell viability, we assessed the effects of 100 and 200 µg/mL MpHE on SN56 cell morphology.

SN56 cells treated with 100 or 200 µg/mL MpHE exhibited a neuronal-like morphology characterized by a centrally located soma and extended neuritic processes (Figure 3A). Cells were classified as branched when they displayed neurites at least 1.5 times longer than the soma diameter [18]. Treatment with 100 µg/mL MpHE increased the percentage of branched cells 3.83-fold compared with controls (*p* = 0.02), and a 4.0-fold increase was observed with 200 µg/mL MpHE (*p* = 0.01) (Figure 3B). These results represent proof-of-concept evidence for a neurotrophic/neuroplastic activity of MpHE constituents on a cholinergic neuronal background. Further experiments are needed to determine if MpHE restores in vivo cholinergic arborization.

We next evaluated LTP, the most reliable molecular correlate of memory, using the FASS-LTP method [4,5,19], which allows assessment of synaptic potentiation in size-gated isolated synaptic terminals by monitoring activity-dependent insertion of GluA1-containing AMPA receptors in postsynapses [20,21] and stabilization of the adhesion protein Nrx1β in presynapses [22,23] following cLTP stimulation (Figure 3C).

Synaptosomes were treated with MpHE (1 mg/mL). Calcein-AM staining confirmed that more than 90% of size-gated synaptosomes remained viable at the conclusion of the FASS-LTP protocol (Figure 3D), indicating that MpHE did not compromise synaptosomal integrity. As expected, synaptosomes from WT mice pre-incubated with either Vh (*p* = 0.0153) or MpHE (*p* = 0.0004) before cLTP induction showed a significant increase in Nrx1β^+^GluA1^+^ double-positive synaptic terminals relative to basal levels, confirming successful cLTP induction (Figure 3E,F). Synaptosomes from Vh-treated 5XFAD mice failed to show this increase following cLTP induction (*p* = 0.2496), indicating impaired synaptic potentiation. In contrast, synaptosomes from MpHE-treated 5XFAD mice (*p* = 0.0004) exhibited a recovery of synaptic potentiation comparable to that of WT synaptosomes (Figure 3E,F). These results demonstrate that MpHE directly restores synaptic LTP in 5XFAD mice at the level of isolated synaptic terminals.

### 2.4. Malva parviflora Hydroalcoholic Extract Ameliorates Cognitive Deficits in 5XFAD Female Mice

Having demonstrated that MpHE treatment partially reverses key AD-related pathological hallmarks, including hippocampal APP and soluble Aβ40–42 accumulation, astrogliosis, systemic amyloidosis, serum IL-18 levels, and synaptic LTP deficits, we investigated whether these molecular improvements translated into functional cognitive recovery.

Working memory deficits were confirmed before treatment initiation. WT females aged 6.5 months displayed normal spontaneous alternation (63.42%), whereas age-matched 5XFAD females showed a significant reduction (56.41%; *p* = 0.0051), confirming established cognitive impairment before treatment (Supplementary Figure S1A).

Following completion of the 20-week MpHE treatment regimen, behavioral performance was assessed across multiple cognitive domains. In the spontaneous Y-maze task, both WT groups, Vh-treated (60.81%) and MpHE-treated (60.82%), exhibited spontaneous alternation rates above 50%, confirming that MpHE did not alter working memory in healthy animals (Figure 4A). Vh-treated 5XFAD females displayed the lowest alternation percentage (48.31%), reflecting impaired hippocampal-cortical connectivity (*p* = 0.0100 vs. WT + Vh). MpHE treatment fully restored spontaneous alternation in 5XFAD females (60.41%), reaching levels comparable to WT controls (*p* = 0.0167 vs. 5XFAD + Vh) (Figure 4A). Representative heat maps and trajectory analyses confirmed efficient sequential arm exploration in WT females; in contrast, Vh-treated 5XFAD females predominantly alternated between arms B and C, but MpHE-treated 5XFAD females recovered an A–B–C exploration pattern comparable to WT animals (Figure 4A,B). Total arm entries and distance traveled did not differ significantly across groups, confirming that locomotor activity was unaffected (Figure 4A,B; Supplementary Figure S1E).

Recognition memory was subsequently evaluated using the novel object recognition (NOR) task. Vh-treated 5XFAD females failed to preferentially explore the novel object at either 1 h or 24 h post-training (Figure 4C,D). WT females receiving Vh also did not show a significant preference for the novel object based on exploration time alone (Figure 4C,D). MpHE treatment significantly restored recognition memory in 5XFAD females, as evidenced by increased exploration time directed toward the novel object at both 1 h (*p* = 0.0014) and 24 h (*p* = 0.0199) post-training (Figure 4C,D).

Analysis of additional exploratory parameters further differentiated group performance. The number of direct nose contacts with the novel object was markedly reduced in Vh-treated 5XFAD females at 1 h (7 contacts; *p* = 0.0207) and 24 h (6 contacts; *p* = 0.0030) relative to WT + Vh mice (16 and 17 contacts, respectively) (Figure 4E,F); MpHE treatment did not restore this parameter. Latency to approach the novel object was significantly prolonged in Vh-treated 5XFAD females compared with WT + Vh (*p* = 0.0014) and WT + MpHE (*p* = 0.0006) groups; MpHE treatment significantly reduced this latency in 5XFAD females (*p* = 0.0131), restoring it to levels comparable with WT controls (Figure 4G). Notably, WT females receiving MpHE exhibited the strongest recognition memory performance across all groups, with longer novel-object exploration times (1 h, *p* = 0.0006); 24 h, *p* = 0.0044), a greater number of novel-object contacts compared with Vh-treated 5XFAD females (1 h, *p* = 0.0037; 24 h, *p* = 0.0338) (Figure 4E,F), and shorter approach latencies (1 h, *p* = 0.0006) (Figure 4G). Representative heat maps and trajectory plots confirmed these quantitative findings. WT + Vh mice preferentially explored the novel object, Vh-treated 5XFAD females exhibited disorganized exploratory behavior with minimal novel-object interaction, and MpHE-treated 5XFAD females recovered a focused exploratory pattern with increased novel-object occupancy and reduced approach latency (Figure 4H). Collectively, these results demonstrate that MpHE treatment shifts the pathological cognitive phenotype of 5XFAD female mice toward a homeostatic, WT-like behavioral state.

## 3. Discussion

Age-related neurodegenerative disorders represent a major global public health challenge, driven in large part by the increase in human life expectancy over recent decades, which has expanded the population susceptible to developing brain disorders [24,25,26]. Pronounced sexual dimorphism has been reported across most neurodegenerative diseases, including Parkinson’s disease, Huntington’s disease, multiple sclerosis, and AD [27]. Despite the fact that women account for approximately two-thirds of all AD cases, most preclinical studies have paradoxically focused on male mouse models. This imbalance may have introduced important biases in our understanding of sex-specific disease mechanisms and therapeutic responses, thereby limiting the translational value of preclinical findings. Emerging research has demonstrated that Aβ burden, tau load, GFAP levels, synaptic loss, and cognitive decline are greater in females than in males with AD, suggesting that sex-specific approaches will be critical to the development of precision pharmacological interventions targeting Aβ clearance, neuroinflammation, synaptic function, and memory [13,28,29,30].

Studies evaluating plant-derived natural products as potential therapeutic alternatives for AD have generated considerable interest owing to their favorable safety profiles; however, these investigations have been conducted predominantly in male models. Among the few AD studies performed in females, one evaluated biochanin A (BCA), a phytoestrogenic flavonoid enriched in red clover, alfalfa, soybean, peanut, and chickpea, in 3-month-old ovariectomized APP/PS1 mice and reported that BCA treatment reduced amyloid burden and β-secretase levels, restored mitochondrial and synaptic function, and rescued associative and spatial memory deficits [31]. The underrepresentation of females in preclinical AD research underscores the importance of incorporating female subjects into both basic and translational neuroscience studies.

Based on this reasoning, and given that MpHE exerts neuroprotective effects under a preventive administration schedule in distinct male models of cognitive impairment [12,32,33], we investigated whether MpHE could reverse AD-related hallmarks specifically in female 5XFAD mice under a therapeutic paradigm. Before treatment initiation, we confirmed that 6.5-month-old female 5XFAD mice exhibited a key AD-associated phenotype, namely impaired working memory, consistent with previous reports of reduced spatial working memory in this model [34], as well as soluble and insoluble amyloid accumulation, glial activation, neuroinflammation, synaptic dysfunction, and behavioral alterations [13,28,29,30]. Female 5XFAD mice aged 6.5 months were then subjected to prolonged therapeutic MpHE administration. In contrast to the preventive effects of MpHE on Aβ plaque reduction and neuroinflammatory suppression observed in male 5XFAD mice [12], therapeutic MpHE administration did not reduce Aβ plaque burden or hippocampal expression of the pro-inflammatory cytokines *il-1β* and *il-6* in female 5XFAD mice. The resistance to plaque clearance and inflammatory resolution likely reflects the higher baseline Aβ burden consistently reported in female 5XFAD mice relative to males; females exhibit a greater number of Aβ plaques in the cortex and hippocampus at 12 months of age, and this difference is already apparent in the cortex at 4 months, suggesting greater pathological severity at early stages in females [35,36]. Furthermore, in the J20 AD model, pharmacological or genetic inhibition of caspase-1 shifted the behavioral phenotype toward a homeostatic state in both female and male adult and aged mice [11,37] without modifying the cytokine profile, although Iba1^+^ microglia and GFAP^+^ astrocyte levels were reduced and Aβ plaque density was partially decreased. Consistent with these observations, multiple studies have demonstrated that therapeutic strategies for AD do not necessarily require reductions in Aβ plaque burden or neuroinflammation to achieve functional recovery [38]. Notably, MpHE treatment reduced both hippocampal and systemic soluble Aβ protein levels, as well as serum levels of the pro-inflammatory cytokines IL-6 and IL-18. Elevated circulating Aβ and IL-18 levels have been reported in both men and women with AD [39,40], and may promote oxidative injury by inhibiting the antioxidant enzymes PRX3 and PRX6, disrupting neuronal metabolism and generating a neurotoxic oxidative environment [41,42,43]. These findings suggest that MpHE constituents may inhibit the amyloidogenic pathway at one or more regulatory points, thereby reducing systemic amyloidosis and systemic inflammation. The reduction in serum IL-18 levels following MpHE treatment provides a mechanistically plausible upstream entry point: IL-18 promotes APP processing and Aβ production through a feedforward loop [8], and its attenuation could account for the observed reduction in soluble Aβ and APP accumulation independently of effects on insoluble plaque burden. This dissociation between soluble and insoluble Aβ species positions the primary action of MpHE at the level of soluble Aβ processing or clearance, consistent with the current consensus that soluble Aβ species are the primary drivers of synaptic dysfunction and cognitive impairment [6,7]. The direct restoration of synaptic LTP in isolated hippocampal synaptosomes upon ex vivo MpHE incubation further demonstrates a cell-autonomous synaptic mechanism of action that operates independently of systemic inflammation, Aβ burden, or astrogliosis, identifying the synaptic terminal as a direct pharmacological target of MpHE bioactive constituents. Further experiments are required to identify the precise molecular target(s) within the synaptic terminal and the upstream receptor through which MpHE modulates IL-18 levels and Aβ processing. To directly test whether MpHE exerts its effects on soluble Aβ through modulation of IL-18 signaling, future experiments should include: (i) IL-18 neutralization assays in which MpHE-treated 5XFAD mice receive a neutralizing anti-IL-18 antibody or recombinant IL-18 binding protein (IL-18BP) to determine whether blocking IL-18 occludes or replicates the effects of MpHE on APP accumulation and soluble Aβ levels; (ii) pathway-specific pharmacological inhibitors targeting the IL-18 receptor signaling cascade, particularly the MyD88–NF-κB axis through which IL-18 drives BACE-1 upregulation and APP processing, administered concomitantly with MpHE to dissect the contribution of this pathway; and (iii) in vitro Aβ production experiments in which SN56 cholinergic cells or primary neurons exposed to IL-18 are co-treated with MpHE or its purified bioactive constituents (scopoletin, oleanolic acid), measuring APP processing products and Aβ40/42 secretion. These experiments would establish whether the reduction in circulating IL-18 observed following MpHE treatment is a cause or a consequence of reduced soluble Aβ, and whether the IL-18/BACE-1/APP axis is a direct pharmacological target of MpHE constituents.

Among the most significant findings of this study is the reduction in GFAP protein levels in the hippocampal region of adult female 5XFAD mice following MpHE treatment, a particularly relevant observation given that higher GFAP levels have been specifically associated with worse global cognition in women compared with men [10,44]. Several studies indicate that modulation of astroglial activation through distinct strategies [4,45] can shift neurotoxic astrocyte phenotypes, including disease-associated astrocytes (DAAs) [46], toward less harmful states, thereby improving learning and memory. Data from our group have previously demonstrated that preventive MpHE administration reduces hippocampal GFAP levels in male 5XFAD mice, an effect consistent with the therapeutic reduction observed here in females.

A prevailing model proposes that in 5XFAD, APP/PS1, and J20 mice, as well as in individuals carrying APP or *PSEN1/2* mutations, Aβ peptides initiate neuroinflammation and glial activation-mediated synaptic damage. Microglia and astroglia are among the most relevant cellular mediators of AD progression. Since microglia are the primary phagocytic cells of the brain, and because MpHE treatment did not reduce insoluble Aβ plaques or hippocampal pro-inflammatory cytokine transcripts in female 5XFAD mice, microglial burden and function were not assessed in the present study; however, a contribution of microglia to the observed effects of MpHE in female 5XFAD mice cannot be excluded.

Astrocytes occupy approximately 20–40% of the total cellular volume of the brain parenchyma [46,47], supporting the view that astrocytes may be as important as microglia in the pathophysiology of brain disorders. Aβ peptides induce astroglial activation in AD [48], shifting homeostatic astrocytes from their physiological roles in metabolic and synaptic support toward a detrimental, neurotoxic state that contributes to behavioral alterations in mouse models and humans [4,45,46,49]. DAAs, enriched in male and female 5XFAD mice and in the brains of AD patients, are characterized by canonical markers including *Gfap*, *Serpina3n*, and *Ctsb*, which are strongly associated with greater Aβ accumulation and increased disease severity [46]. The MpHE-induced reduction in GFAP^+^ astrocytes is therefore consistent with a possible shift away from the DAA phenotype, a hypothesis that requires direct transcriptomic and functional characterization of astrocyte subpopulations to be confirmed. Notably, *Ctsb*, encoding cathepsin B, has been implicated in excessive APP processing in neurological disorders [50]. The significant reduction in hippocampal APP accumulation observed in MpHE-treated female 5XFAD mice may therefore reflect reduced astroglial reactivity and potentially lower cathepsin B activity, a mechanistic hypothesis that warrants direct experimental evaluation.

Consistent with these findings, Boers-Escuder et al. [51] recently demonstrated that deletion of the astroglial genes *Gfap* and *Vimentin* in the APP/PS1 mouse model produced cognitive recovery remarkably similar to that observed in MpHE-treated female 5XFAD mice, accompanied by restoration of synaptic markers and proteins involved in cellular translation and, importantly, these effects were maintained in the absence of changes in insoluble amyloid burden or microglial reactivity. Taken together with the present findings, these studies suggest that modulating astroglial activity, whether genetically or pharmacologically, promotes a neuroprotective phenotype across distinct AD mouse models.

Loss of dendritic arborization, reduced synaptic function, and impaired neuronal connectivity in the hippocampus and cortex are major hallmarks of AD that underlie the deterioration of higher-order cognitive processes, including learning, memory, decision-making, and executive function [4,5,35,52]. Given that preventive MpHE treatment in male 5XFAD mice rescues Aβ-induced spatial learning and memory deficits [12], and that dendritic complexity is associated with functional synaptic connectivity, we evaluated whether MpHE promotes basal neurite outgrowth in the SN56 cholinergic neuronal cell line, which originates from the septal basal forebrain and exhibits a well-characterized cholinergic phenotype [52,53]. This model is particularly relevant because acetylcholine levels and signaling are impaired in AD mouse models and in individuals with the disease [54,55]. At both tested concentrations, MpHE significantly increased the percentage of SN56 cells displaying extensive neuritic branching in vitro, providing proof-of-concept evidence for a neurotrophic activity of MpHE constituents on a cholinergic cellular background. The translational significance of these findings to in vivo cholinergic arborization cannot be directly inferred from the cell line data and requires confirmation in primary cholinergic neurons or in vivo morphological analyses. Consistent with these morphological findings, pre-incubation of hippocampal synaptosomes from adult female 5XFAD mice with MpHE prior to chemical LTP induction restored synaptic potentiation to levels comparable to those of WT synaptosomes. These findings suggest that MpHE constituents, including oleanolic acid and scopoletin, may act directly at the neuronal and synaptic level to promote restoration of synaptosomal signaling in the 5XFAD background. In support of this interpretation, Hornick et al. [56] demonstrated that pre-incubation of rat cortical synaptosomes with scopoletin enhanced activity-dependent acetylcholine release and improved hippocampal CA1 LTP relative to controls, and that scopoletin improved memory in 15–18-month-old mice with age-associated cognitive deficits. Given that scopoletin promotes activity-dependent acetylcholine release and that cholinergic signaling is compromised in both AD mouse models and patients, scopoletin may contribute to restoration of cholinergic function in adult female 5XFAD mice, a hypothesis that should be evaluated experimentally ex vivo and in vivo. This is further supported by the fact that several approved AD therapies are designed to restore acetylcholine levels and function, given the essential role of this neurotransmitter in neuronal plasticity and cognition.

MpHE treatment restored hippocampus-dependent working memory in adult female 5XFAD mice relative to Vh-treated transgenic controls. Given that working memory is integrated across hippocampal–cortical circuits, these data suggest that MpHE recovers functional connectivity among multiple brain regions. The magnitude of memory rescue was comparable to that previously reported under the preventive treatment paradigm in male 5XFAD mice [12]. Importantly, no differences were observed in total arm entries or distance traveled during the Y-maze task, indicating that recovery of working memory reflects improved hippocampal function rather than altered locomotor activity.

In the NOR task, MpHE-treated 5XFAD mice showed restored novel-object exploration time during both short- and long-term memory phases. An unexpected observation was the absence of a significant preference for the novel object based on exploration time in the WT + Vh group. The limited availability of studies in adult or aged female mice constrains direct comparison with the existing literature; however, age, genetic background, and sex may collectively modify the innate tendency to explore novel stimuli. Female WT mice at 11.5–12 months of age may undergo more pronounced age-related changes than males, resulting in reduced exploration time owing to altered neurotransmitter levels, disrupted interregional connectivity, or age-associated basal inflammation on the C57BL/6J × SJL background. Notably, MpHE administration increased short- and long-term novel-object exploration time in WT females, suggesting that MpHE metabolites may exert neuroprotective effects during normal female aging, a hypothesis that warrants dedicated sex-specific investigation.

Exploratory behavior in the NOR task should not be evaluated through interaction time alone. Additional parameters, including the number of nose contacts with the novel object and the latency to approach it, provide complementary information [57]. In the present study, the innate exploratory response, as measured by the number of nose contacts, was impaired in Vh-treated 5XFAD females and was not restored by MpHE treatment. In contrast, the latency to approach the novel object was significantly prolonged in 5XFAD + Vh mice and was reduced by MpHE treatment to levels comparable with WT controls. Taken together, these data suggest a partial loss of exploratory behavior in adult WT females, while MpHE prolongs and facilitates interaction with novel stimuli in both WT and 5XFAD genotypes. Importantly, MpHE produced a marked rescue of associative memory in 5XFAD females, with interaction time and approach latency closely resembling those of MpHE-treated WT mice, a behavioral recovery that correlates with the synaptic rescue observed in hippocampal synaptosomes from adult female 5XFAD mice.

Considered together, although WT + Vh females did not exhibit a significant preference for the novel object based on exploration time, they displayed more nose contacts and a shorter approach latency than 5XFAD + Vh mice, suggesting preserved novelty detection and attentional orientation in the absence of sustained recognition memory preference. MpHE-treated female 5XFAD mice, in contrast, exhibited preserved hippocampal integrity and function alongside substantial improvement in recognition memory. The observation that MpHE restored exploration time directed at the novel object but did not recover the absolute number of nose contacts deserves specific consideration. These two parameters reflect partially dissociable behavioral processes: exploration time indexes the capacity to sustain interest in a recognized novel stimulus, a measure of recognition memory, whereas nose contact frequency reflects the initial intensity of the novelty-driven exploratory impulse, which may depend on olfactory processing, attention allocation, and motivational circuits that are partially independent of hippocampal mnemonic function. This pattern is consistent with a partial functional recovery in which hippocampal-dependent recognition memory is restored, whilst circuits governing motivational and attentional aspects of exploratory behavior, including dopaminergic and cholinergic inputs to the prefrontal cortex, may require additional or complementary therapeutic targeting. Future studies evaluating whether extended treatment duration or combination with cholinesterase inhibitors further recovers nose contact frequency would clarify the ceiling of MpHE-mediated cognitive rescue in this model. These findings position MpHE constituents as promising candidates for therapeutic development in AD, as they may exert hippocampal neuroprotective effects and downregulate classical pathology markers, shifting cognition toward a functionally resilient phenotype despite persistent insoluble amyloid burden. A methodological consideration that warrants acknowledgment is the relationship between the in vivo dose employed (50 mg/kg/day, intragastric) and the in vitro concentrations used in the FASS-LTP synaptosomal assay (1 mg/mL) and the SN56 cell morphology experiments (100–200 µg/mL). Direct pharmacokinetic equivalence between these experimental systems cannot be established without bioavailability, absorption, and tissue distribution data for MpHE and its individual bioactive constituents in the mouse. The in vitro concentrations were selected based on prior studies demonstrating anti-inflammatory effects of MpHE within a range of 100–1000 µg/mL without compromising cell viability [12,32], and the synaptosomal concentration of 1 mg/mL was chosen to ensure sufficient extract availability in the extracellular milieu of isolated terminals under ex vivo conditions. Whether these concentrations are achievable in hippocampal tissue following oral intragastric administration at 50 mg/kg remains to be determined through pharmacokinetic profiling studies. Notably, scopoletin, a key bioactive constituent of MpHE, is known to cross the blood–brain barrier and accumulate in brain tissue at biologically active concentrations following systemic administration [56]; however, systematic pharmacokinetic characterization of MpHE as a whole extract in the 5XFAD model is a recognized limitation of the present study and a necessary step toward translational development. Recent findings by Ouyang and colleagues demonstrated that scopoletin treatment restores cognitive function in both female and male 5XFAD mice [57], consistent with the neuroprotective phenotype observed here in MpHE-treated female 5XFAD mice and suggesting that the beneficial effects of MpHE may be attributable, at least in part, to its scopoletin content.

The observation that MpHE improves synaptic function and cognitive recovery is particularly compelling because it resembles the phenotype of cognitively resilient individuals with AD, characterized by a high cerebral Aβ burden and reduced reactive gliosis while preserving synaptic integrity and function, thereby maintaining intact cognition throughout life despite substantial neuropathology [58,59]. Our findings therefore raise the possibility that MpHE treatment promotes a pattern of pathological and functional findings consistent with an AD-resilient phenotype, supporting the view that synaptic dysfunction, likely astrocyte-mediated, represents the closest functional correlate of cognitive impairment in AD. However, additional analyses including characterization of insoluble plaque morphology, dendritic number and complexity, and astrocyte transcriptomic profiles will be required to test this hypothesis. A primary limitation of the present study is the absence of male mice, which prevents direct within-study sex comparison and precludes any conclusion regarding sex-specificity of the observed effects. The present investigation was designed as a deliberate complement to our prior work in male 5XFAD mice under a preventive paradigm [12]; together, the two studies address both sexes and both intervention schedules. Nevertheless, whether the effects of MpHE on soluble Aβ levels, astrogliosis, IL-18 signaling, cholinergic neuronal branching, and synaptic LTP restoration are specific to females or are shared across sexes remains an open question that future studies including both sexes under the therapeutic paradigm must address. It should also be acknowledged that the absence of MpHE effects on insoluble Aβ plaque burden and hippocampal inflammatory cytokine expression does not exclude the possibility that a higher dose, an alternative route of administration, or a longer treatment duration could produce effects on these endpoints. The dose of 50 mg/kg/day and the 20-week treatment period were selected based on prior efficacy and safety data from our group [12,32]; however, formal dose–response studies under the therapeutic paradigm have not been conducted, and the question of whether plaque clearance and transcriptional anti-inflammatory effects of MpHE are achievable at higher doses in established disease remains open.

The absence of systemic toxicity following prolonged MpHE administration was evaluated through behavioral, biochemical and histological analyses (Supplementary Figure S3). At the end of the treatment period, animals were subjected to the open field test to see changes in their exploration and motor activity. Traversed distance, average speed, time spent at the center and grooming and rearing frequency were measured, finding no differences between the MpHE-treated and the control group in any of these parameters (Supplementary Figure S3A–E), indicating no deleterious effect on exploration and motor activity from the MpHE chronic administration. Besides this, no apparent differences were seen in animal tracks between the groups (Supplementary Figure S3F). Furthermore, motor coordination and balance were evaluated through the Rota-rod test, showing a decrease in the fall latency as the rotor speed increased, but presenting no changes between the MpHE- and vehicle-treated animals (Supplementary Figure S3G). Presuming no deleterious effect of the MpHE chronic administration on mice central nervous system. Besides this, renal function was assessed by measuring urinary protein, creatinine clearance, and the urine protein-to-creatinine ratio (UPCR); no urinary protein was detected, and both creatinine clearance and UPCR remained unchanged (Supplementary Figure S3H–J). Hepatic function was evaluated by measuring serum transaminase activities; glutamic-oxaloacetic transaminase (GOT) and glutamic-pyruvic transaminase (GPT) did not differ between MpHE- and Vh-treated animals (Supplementary Figure S3K,L). Histological evaluation of kidney and liver tissue by hematoxylin and eosin staining revealed no evidence of glomerular alterations, tubular damage, parenchymal inflammation, necrosis, fibrosis, or steatosis in either organ. These findings indicate that chronic MpHE administration does not compromise essential systemic physiological functions, an observation of particular relevance when compared with currently approved AD therapies such as donepezil, rivastigmine, and galantamine, which produce only partial improvements in memory and are associated with prominent adverse effects including cardiac complications, gastrointestinal disturbances, nausea, diarrhea, and weight loss with long-term use [60,61]. During MpHE treatment, no weight loss, diarrhea, or abnormal stool consistency was observed; nevertheless, additional studies are required to establish the cardiovascular and gastrointestinal safety profile of MpHE. A significant translational limitation is the chemical complexity of MpHE as a hydroalcoholic plant extract. While we have previously characterized the presence of scopoletin and oleanolic acid as bioactive constituents [12], the extract contains a diversity of flavonoids, phenolic acids, and other phytochemicals whose individual contributions to the observed effects on soluble Aβ, astrogliosis, IL-18, synaptic LTP, and cognitive function cannot be determined from the present data. The observed effects may result from a single dominant bioactive constituent, from additive contributions of multiple components, or from synergistic interactions among extract constituents. Systematic fractionation studies, followed by bioactivity-guided isolation and testing of individual fractions and purified compounds in the therapeutic 5XFAD paradigm, are required to identify the active principle(s) and to progress toward a defined pharmacological entity suitable for translational development.

Finally, recent evidence suggests that the postmenopausal ovary may acquire an inflammatory and senescence-like phenotype in older women [62]. This observation is particularly relevant because ovariectomy accelerates several AD-related hallmarks in mouse models [30,63] and has been associated with a higher incidence of cognitive deficits and neurodegeneration in women [64,65]. Given the greater predisposition of women to develop AD and the possibility that age-related ovarian dysregulation may represent an important component of AD pathophysiology, and given that structurally related flavonoids present in MpHE have been shown to modulate female hormonal pathways in silico analyses conducted in our group, we propose that MpHE may regulate the cellular and molecular phenotype of the ovary and thereby contribute to protection of the brain from AD-related pathology. Further experiments are required to test this hypothesis.

## 4. Materials and Methods

### 4.1. Animals and MpHE Treatment

Adult female 5XFAD transgenic mice expressing five familial AD mutations, three in the *APP* gene (Swedish: K670N/M671L; Florida: I716V; London: V717I) and two in the *PSEN1* gene (M146L and L286V), on a C57BL/6J × SJL genetic background, and wild-type (WT) C57BL/6J × SJL littermates, were obtained from The Jackson Laboratory (Bar Harbor, ME, USA). Mice were housed in groups of 3–5 animals per cage in a specific pathogen-free animal facility under standard conditions at temperature (25 °C)- and humidity-controlled room on a 12 h light/dark cycle with food and water available *ad libitum*. Genotypes were confirmed by PCR. All animal procedures were approved by the Ethics and Institutional Bioethics Committee of the Universidad Nacional Autónoma de México under project number 524 and adhered to the ARRIVE guidelines.

The sample size (n) per group was determined based on a previous study conducted by our group in male mice undergoing behavioral assessments (n = 8) [12]. Given the limited available literature on female mice at the ages examined in the present study, the sample size was increased to 14–19 female mice per group to improve the statistical power and robustness of the behavioral analyses. We used 31 total 5XFAD and 37 WT mice littermates aged 6.5–11.5 months that received daily 50 mg/kg/day of MpHE or water (as vehicle) by intragastric administration for 20 weeks. The exact number of animals used in each experiment is indicated in the graphs; each dot represents one mouse.

Animals were randomly assigned to experimental groups using a randomization scheme that ensured littermates were distributed across all four groups (WT + Vh, WT + MpHE, 5XFAD + Vh, 5XFAD + MpHE). Randomization was performed at 6.5 months of age. All exclusion criteria were established a priori. Animals showing signs of illness unrelated to genotype at the start of the experiment were excluded prior to group assignment. Animals that did not meet the minimum locomotor activity threshold (total arm entries ≥ 5) in the Y-maze spontaneous alternation task were excluded from behavioral analysis of that specific test. Animals showing a total object exploration time during the training session <5 s in the NOR task were excluded. Analysis was performed in a blind manner. Blinding was maintained until after all quantifications were complete.

The dose of 50 mg/kg/day was selected based on our previously published dose-finding work demonstrating that this dose produces anti-inflammatory and neuroprotective effects in male 5XFAD mice without evidence of systemic toxicity and is consistent with effective doses reported for *Malva parviflora* extracts in other murine inflammatory and neurodegenerative models [12,32]. Formal dose–response studies under the current therapeutic paradigm—initiated after established AD pathology—have not been conducted, and it is acknowledged that higher or lower doses may produce different activity profiles on the endpoints examined in the present study. Such studies are planned as a next experimental step. The aerial parts of Malva parviflora were obtained in a single collection at Ozumba, state of Mexico, Mexico. One specimen was identified in the herbarium of the Ethnobotanical Garden of the Museum of Traditional Medicine and Herbal Medicine of INAH Morelos with the registration number 2048. Preparation of MpHE and storage conditions have been described previously [12].

The animals were consistently housed in the same room within the animal facility. Animal cages were labeled with their genotype and treatment throughout received daily doses adjusted according to their body weight, with all administrations performed by the same experimenter.

### 4.2. Protein Extraction and Immunoblotting

Total protein was extracted from hippocampal tissue using lysis buffer (20 mM Tris pH 7.4, 137 mM NaCl, 25 mM β-glycerophosphate, 2 mM sodium pyrophosphate, 2 mM EDTA, 1% Triton X-100, 10% glycerol) supplemented with 1 mM PMSF, 0.5 mM DTT, 200 mM Na_3_VO_4_, and a cOmplete^TM^ protease inhibitor cocktail (Roche, Basel, Switzerland). Tissue was minced and sonicated. Lysates were incubated for 15 min at 4 °C and centrifuged at 14,000 rpm for 10 min at 4 °C. Supernatants were collected and stored at −70 °C. Protein concentration was determined using the Pierce™ BCA Protein Assay Kit (Thermo Fisher Scientific, Waltham, MA, USA).

Twenty micrograms of protein per sample were resolved by SDS-PAGE and transferred onto nitrocellulose membranes. Membranes were blocked with 5% non-fat dry milk in TBS-T (20 mM Tris pH 7.5, 150 mM NaCl, 0.1% Tween-20) for 1 h at room temperature, then incubated overnight at 4 °C with the corresponding primary antibodies: anti-GFAP (1:2000; Chemicon, Temecula, CA, USA, Cat. AB5541, RRID: AB_177521); anti-Aβ (1:3000; BioLegend, San Diego, CA, USA, Cat. SIG-39320, RRID: 662798) to recognized full APP and Aβ peptides; and anti-GAPDH (1:10,000; Cell signaling, Danvers, MA, USA, Cat. 2118S, RRID: AB_561053). Heat-induced epitope retrieval was performed before incubation with the anti-Aβ antibody. HRP-conjugated secondary antibodies were incubated for 2 h at room temperature. Proteins were detected using ECL Plus (PerkinElmer, Waltham, MA, USA) according to the manufacturer’s instructions and visualized using a C-DiGit Blot Scanner (LI-COR, model 3600). Densitometric analysis was performed using Image Studio Software version 5.5 (LI-COR Biosciences, Lincoln, NE, USA). Protein levels were normalized to GAPDH, and the relative protein level of the WT Vh group was set to 1.

### 4.3. Thioflavin S Staining

5XFAD and WT female mice were anesthetized with an intraperitoneal injection of ketamine/xylazine (100 mg/kg/10 mg/kg) and transcardially perfused with 0.9% saline solution followed by 4% paraformaldehyde (PFA) in phosphate-buffered saline (PBS, pH 7.4). Brains were harvested, post-fixed in 4% PFA for 48 h, and cryoprotected in 30% sucrose (*w*/*v*) in PBS containing 0.01% sodium azide. Serial sagittal brain sections (30 µm) were collected spanning 0.36–3.00 mm lateral to bregma.

Sections were washed with Tris-buffered saline (TBS) and stained with 0.25% Thioflavin S for 5 min, followed by sequential washes with graded ethanol (80% for 30 s, then 70% for 3 min) to remove unbound dye, and rinsed with distilled water. Nuclear counterstaining was performed with DAPI (1:12,000; Molecular Probes, Eugene, OR, USA, Cat. D1306) for 15 min. Sections were mounted with Fluoroshield (Sigma-Aldrich, St. Louis, MO, USA, Cat. F6182). Confocal imaging of hippocampal Aβ plaques was performed using a 2 × 3 mosaic configuration on a Marianas spinning-disk confocal system (3i, Inc., Denver, CO, USA) with a 63× objective and emission at 550 nm. Images were acquired using SlideBook 6.0 software (3i, Inc.); signal acquisition parameters were standardized to prevent saturation and optimize the signal-to-noise ratio.

### 4.4. Real-Time Quantitative RT-PCR

Total RNA was isolated from hippocampal tissue using TRIzol reagent (Thermo Scientific) according to the manufacturer’s instructions. RNA concentration was quantified using a NanoDrop 2000 spectrophotometer (Thermo Scientific, Waltham, MA, USA). Five hundred nanograms of total RNA were reverse-transcribed using the RevertAid First Strand cDNA Synthesis Kit (Thermo Fisher Scientific) with random hexamers, following the manufacturer’s guidelines.

Quantitative PCR was performed using Maxima SYBR Green/ROX Master Mix (Thermo Fisher Scientific) with 100 nM of gene-specific primers at an annealing temperature of 64 °C on a StepOne Real-Time PCR System (Applied Biosystems, Foster City, CA, USA). Gene expression was normalized to *Actb,* and relative quantification was calculated using the 2^−ΔΔCT^ method. Forward (F) and reverse primers used: For *Tnf*: F 5′-GGA TGA GAA GTT CCC AA ATG-3′ and R 5′-CACTTGGTGGTTTGCTACG-3′; for *Il-1β*: F 5′-GAATGACCTGTTCTTTGAAGTTG-3′ and R 5′-CTTCTCCACAGCCACAATG-3′; for *Il-6*: F 5′-GAACAACGATGATGCACTTG-3′ and R 5′-GGTACTCCAGAAGACCAGAGG-3′; and for *Actb:* F 5′-CTAAGGCCAACCGTGAAAAG-3′ and R 5′-CATCACAATGCCTGTGGTAC-3′.

### 4.5. Enzyme-Linked Immunosorbent Assay (ELISA)

Serum Aβ42 concentrations were determined using the Mouse Aβ42 ELISA Kit (Invitrogen, Waltham, MA, USA, Cat. KMB3441) following the manufacturer’s instructions. Briefly, 10 µL of serum was diluted in 90 µL of standard diluent buffer before analysis.

### 4.6. Cytokine Measurements

Serum levels of CXCL1, TGF-β1 (free active form), IL-18, IL-23, CCL22 (MDC), IL-10, IL-12p70, IL-6, TNF-α, G-CSF, CCL17 (TARC), IL-12p40, and IL-1β were quantified in WT and 5XFAD female mice using a multi-analyte bead-based flow assay (Mouse Macrophage/Microglia Panel 13-plex, BioLegend, Cat. 740845) according to the manufacturer’s guidelines. Serum samples and mixed beads were incubated at 750 rpm for 3 h, washed, and incubated for 1 h with biotinylated detection antibody. Streptavidin-phycoerythrin was added without washing and incubated for 30 min. Samples were washed and acquired on a FACSCanto II cytometer (BD Biosciences, San Jose, CA, USA) using FACSDiva software (version 9.0.1), recording 5000 events at a low flow rate. Data analysis was performed using LEGENDplex™ cloud-based software (legendplex.qognit.com (accessed on 2 August 2026), version 2021-04-15).

### 4.7. SN56 Cells and MpHE Treatment

The SN56 cholinergic neuronal cell line, kindly provided by Prof. Bruce Wainer (Department of Pathology, University of Chicago, Chicago, IL, USA) [66,67], was maintained in Advanced DMEM supplemented with 2% fetal bovine serum (FBS), 2 mM L-glutamine, 100 U/mL penicillin, and 50 µg/mL streptomycin at 37 °C in a 5% CO_2_ atmosphere.

4 × 10^4^ SN56 cells were seeded in 6-well plates and treated with MpHE (100 or 200 µg/mL) or vehicle in serum-free Advanced DMEM. After 72 h, microphotographs of five randomly selected fields were acquired at 10× magnification using a Nikon Eclipse TS100 (Nikon, Tokyo, Japan) optical microscope equipped with a Canon Rebel T7 camera (Canon, Tokyo, Japan). The percentage of cells exhibiting neuritic branching was determined relative to the total cell population. Cells were classified as branched when presenting at least one neurite with a length exceeding 1.5 times the diameter of the cell soma. Quantification was performed using the Cell Counter plugin in Fiji ImageJ 2.9.

### 4.8. Fluorescence Analysis of Single-Synapse Long-Term Potentiation (FASS-LTP)

FASS-LTP was conducted as previously described [4,5,19]. Briefly, hippocampi were dissected on ice and homogenized in 1.5 mL of ice-cold 320 mM sucrose solution (pH 7.4) containing 10 mM HEPES and supplemented with cOmplete™ Protease Inhibitor Cocktail (Roche). Homogenization was performed by manually rotating a Teflon pestle 6–8 times in a pre-cooled glass Dounce homogenizer (clearance 0.15–0.25 mm) to minimize mechanical damage to synaptosomes. The homogenate was centrifuged at 1200× *g* for 10 min at 4 °C; the resulting supernatant (S1) was transferred to two ice-cooled tubes and centrifuged at 12,000× *g* for 20 min at 4 °C. The supernatant (S2) was discarded and the pellet (P2, synaptosomal-enriched crude fraction) was resuspended for 1 min in either 1.5 mL of basal solution (120 mM NaCl, 3 mM KCl, 2 mM CaCl_2_, 2 mM MgCl_2_, 15 mM glucose, 15 mM HEPES, pH 7.4) or Mg^2+^-free cLTP solution (125 mM NaCl, 2 mM CaCl_2_, 5 mM KCl, 10 mM HEPES, 30 mM glucose, pH 7.4). The P2 fraction was then allowed to recover for 15 min at room temperature with gentle agitation in a 30 mm Petri dish.

For cLTP induction, 180 µL of P2 fraction in either basal or cLTP solution was pre-incubated with 1 mg/mL MpHE or Vh for 20 min at 37 °C. P2 fractions in cLTP solution then received 20 µL of glycine (final concentration: 500 µM) and were incubated for 15 min, followed by 100 µL of KCl solution (50 mM NaCl, 2 mM CaCl_2_, 100 mM KCl, 10 mM HEPES, 30 mM glucose, 0.5 mM glycine, 0.001 mM strychnine, 0.02 mM bicuculline, pH 7.4) for 30 min. P2 fractions maintained in basal solution received equivalent volumes of basal solution in place of glycine and KCl and served as controls for baseline GluA1^+^Nrx1^+^ levels. cLTP induction was terminated by adding 0.5 mL of ice-cold 0.1 mM EDTA-PBS (pH 7.4) and 4 mL of FACS buffer (PBS with 5% FBS). The P2 fraction was centrifuged at 2500× *g* for 5 min at 4 °C, and the pellet was resuspended.

Immunolabeling was performed in SET buffer (320 mM sucrose, 1 mM EDTA, 5 mM Tris, pH 7.3) to prevent synaptosomal aggregation [68]. P2 fractions were incubated with anti-GluA1 (1:800; Cell Signaling Technology, Cat. 13185, RRID: AB_2732897) and anti-Nrx1 (1:400; Antibodies Incorporated, Davis, CA, USA, Cat. 75-216, RRID: AB_2155531) for 30 min at 4 °C, washed, and centrifuged at 2500× *g* for 5 min. Pellets were incubated with fluorescent secondary antibodies for 30 min at 4 °C in the dark, washed, and centrifuged as above. Synaptosomes were acquired immediately after labeling.

Synaptosomal integrity was assessed in a parallel aliquot of the P2 fraction treated under identical conditions using 100 nM Calcein-AM. Synaptosomes were acquired on a FACSCanto II cytometer using FACSDiva software. Size-gated synaptosomes were defined using calibration beads (Polysciences, Inc., Warrington, PA, USA, Cat. 19822) including 1–3 µm particles and excluding 0.5 µm particles. Ten thousand events were recorded, and analysis was performed on singlets using FlowJo version 10.8.1.

### 4.9. Behavioral Experiments

Behavioral testing was performed in female 5XFAD transgenic mice and WT littermates aged 6.5–11.5 months, with and without MpHE treatment, in a sound-isolated room exclusive for behavioral assessment. Mice were assigned to the treatment group manually through a random draw. Prior to the test, mice were habituated in the test room for 30 min. Data were analyzed using ANY-Maze software version 7.4 (Stoelting Co., Wood Dale, IL, USA). All exclusion criteria were established a priori, and analysis was performed in a blind manner. The experimenter did not have knowledge of the genotype.

### 4.10. Y-Maze Test

Spatial working memory was assessed by the spontaneous alternation Y-maze test. Each animal was placed in a Y-shaped maze and allowed to explore freely for 8 min. The number of arm entries and the sequence of entries were recorded. The spontaneous alternation percentage was calculated as:

$$Alternation\;(\%)=\left[\left.\frac{Number\;of\;correct\;triads}{\left(Total\;entries-2\right)}\right]\right.\times 100$$

A triangle was placed at the center to delimit the origin of the three arms (A, B, and C); the center zone was excluded from quantification. Entry was recorded only when all four limbs were within an arm, excluding the tail. Animals that entered all arms fewer than 5 times during the entire trial were discarded from the analysis. One WT mouse and one 5XFAD mouse in the Y maze test were excluded because they did not meet our inclusion criteria (WT: n = 36; 5XFAD: n = 30; Supplementary Figure S1A–C). We did not exclude mice in Figure 4A since all of them met our inclusion criteria.

### 4.11. Novel Object Recognition Test

Episodic-like memory was assessed using the novel object recognition (NOR) test. Mice were placed in a black wooden open-field box (33 × 33 × 30 cm). The protocol consisted of three phases: a training phase and two test phases at 1 h and 24 h post-training. During training, each mouse explored two identical objects for 5 min. Animals that explored < 5 s during the training session were excluded [5]. During each test phase, one object was replaced with a novel object, and exploration was recorded for 5 min. The box and objects were cleaned with 70% ethanol between animals, and bedding was replaced between subjects to control for olfactory cues. Interaction time within 1 cm of each object, the number of direct nose/object contacts, and latency to first contact with the novel object were quantified. Two mice from the WT + Veh group; two mice from the WT + MpHE group; two mice from the 5XFAD + Vh group; and four mice from the 5XFAD + MpHE group were excluded as they had a total object exploration time during the training session < 5 s.

### 4.12. Open Field Test

Motor function and anxiety-like behavior were assessed in a 50 × 50 × 50 cm plexiglass open-field arena over a 5 min session without prior habituation. The arena was divided into a center zone and a peripheral zone. Time spent in the center, number of center crossings, distance traveled in the center, latency to first center entry, and total distance and mean speed were quantified.

### 4.13. Statistics

All data are presented as the mean ± standard error of the mean (SEM). Sample size was selected based on our preliminary studies [12]. Prior to statistical evaluation, normal distribution and homogeneity of variances were assessed using the Shapiro–Wilk test and Brown-Forsythe test (or Levene’s), respectively. For comparison experiments of the βA levels, we used 5XFAD mice treated with vehicle as a control and used a two-tailed unpaired Student’s *t*-test, whereas for comparisons of more than two groups with a single independent variable we used One-way ANOVA followed by Tukey’s or the two-stage linear step-up procedure of Benjamini, Krieger, and Yekutieli to control the false discovery rate (FDR). For NOR experiments, preference or exploration times within groups were analyzed using two-tailed paired or unpaired Student’s *t*-tests as appropriate, whereas inter-group comparisons for approach latencies, number of contacts, and Y-maze alternation percentages were analyzed using one-way ANOVA followed by Tukey’s post hoc test. Statistical significance was defined as *p* ≤ 0.05. All statistical analyses and graphics were performed using GraphPad Prism software (version 9.0 or higher, GraphPad Software, San Diego, CA, USA).

## Figures and Tables

**Figure 1:**
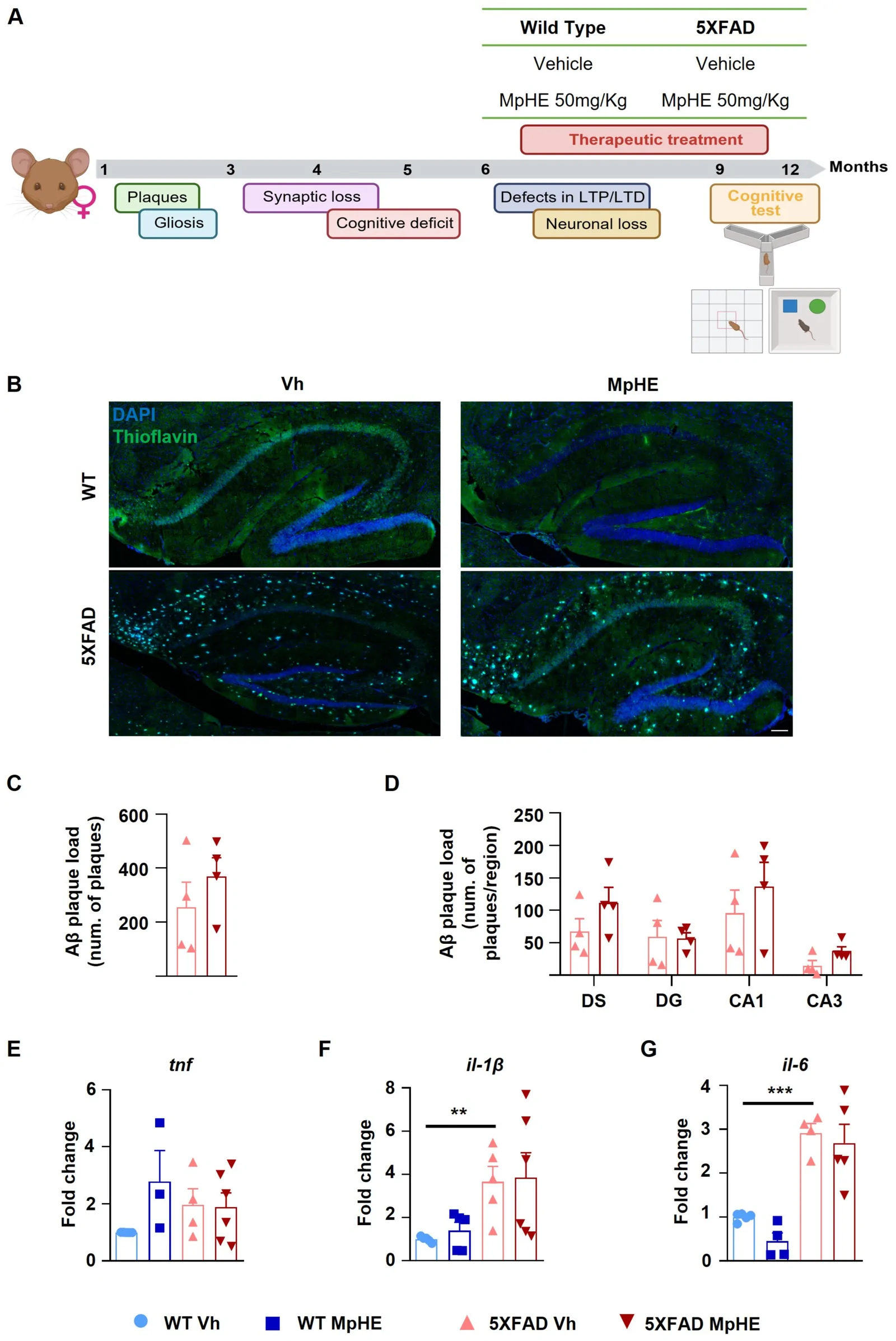
Therapeutic administration of *Malva parviflora* hydroalcoholic extract does not modify amyloid plaque burden or hippocampal inflammatory gene expression in female 5XFAD mice. (**A**) Experimental design. (**B**) Representative confocal microphotographs of Thioflavin S-labeled Aβ plaques in sagittal brain sections from 12-month-old WT and 5XFAD female mice treated with vehicle (Vh) or MpHE. DAPI was used as a nuclear counterstain. Scale bar = 100 µm. (**C**) Total Aβ plaque count in the hippocampus (2 sections per mouse; 5XFAD Vh: n = 4; 5XFAD + MpHE: n = 4). (**D**) Regionalized Aβ plaque count in the dorsal subiculum (DS), dentate gyrus (DG), CA1, and CA3. (**E**–**G**) Relative mRNA expression of *tnf* (WT Vh: n = 5; WT + MpHE: n = 3; 5XFAD Vh: n = 4; 5XFAD + MpHE: n = 6) (**E**), *il-1β* (WT Vh: n = 5; WT + MpHE: n = 5; 5XFAD Vh: n = 5; 5XFAD + MpHE: n = 6) (**F**), and *il-6* (WT Vh: n = 5; WT + MpHE: n = 4; 5XFAD Vh: n = 4; 5XFAD + MpHE: n = 5) (**G**) in hippocampal tissue from WT and 5XFAD female mice treated with Vh or MpHE, determined by RT-qPCR. All data are presented as mean ± SEM. Statistical significance was assessed by one-way ANOVA; ** *p* ≤ 0.005, *** *p* ≤ 0.001.

**Figure 2:**
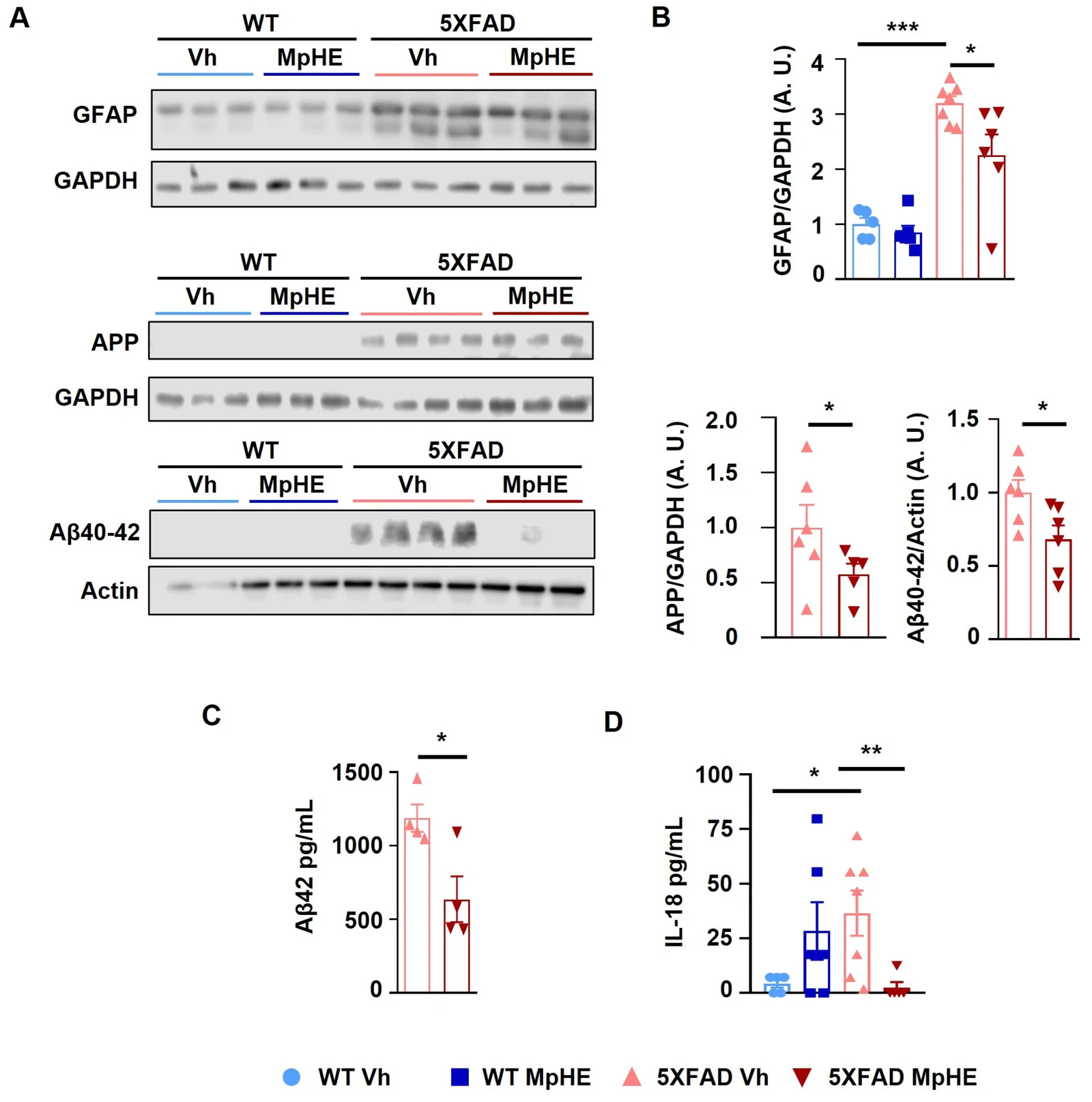
*Malva parviflora* hydroalcoholic extract reduces soluble amyloid-β peptide levels and attenuates astrogliosis in female 5XFAD mice. (**A**) Representative immunoblots of GFAP, APP, and soluble Aβ40–42 monomer in hippocampal tissue from WT and 5XFAD female mice treated with Vh or MpHE. (**B**) Densitometric quantification of immunoblots normalized to GAPDH and actin as loading controls (WT + Vh: n = 5; WT + MpHE: n = 6; 5XFAD + Vh: n = 5; 5XFAD + MpHE: n = 7). (**C**) Serum Aβ42 concentration and (**D**) serum IL-18 concentration in WT and 5XFAD female mice treated with Vh or MpHE, determined by ELISA and multi-analyte bead-based flow assay, respectively. All data are presented as mean ± SEM. Statistical significance was assessed by Student’s *t*-test and one-way ANOVA; * *p* ≤ 0.05, ** *p* ≤ 0.005, *** *p* ≤ 0.001.

**Figure 3:**
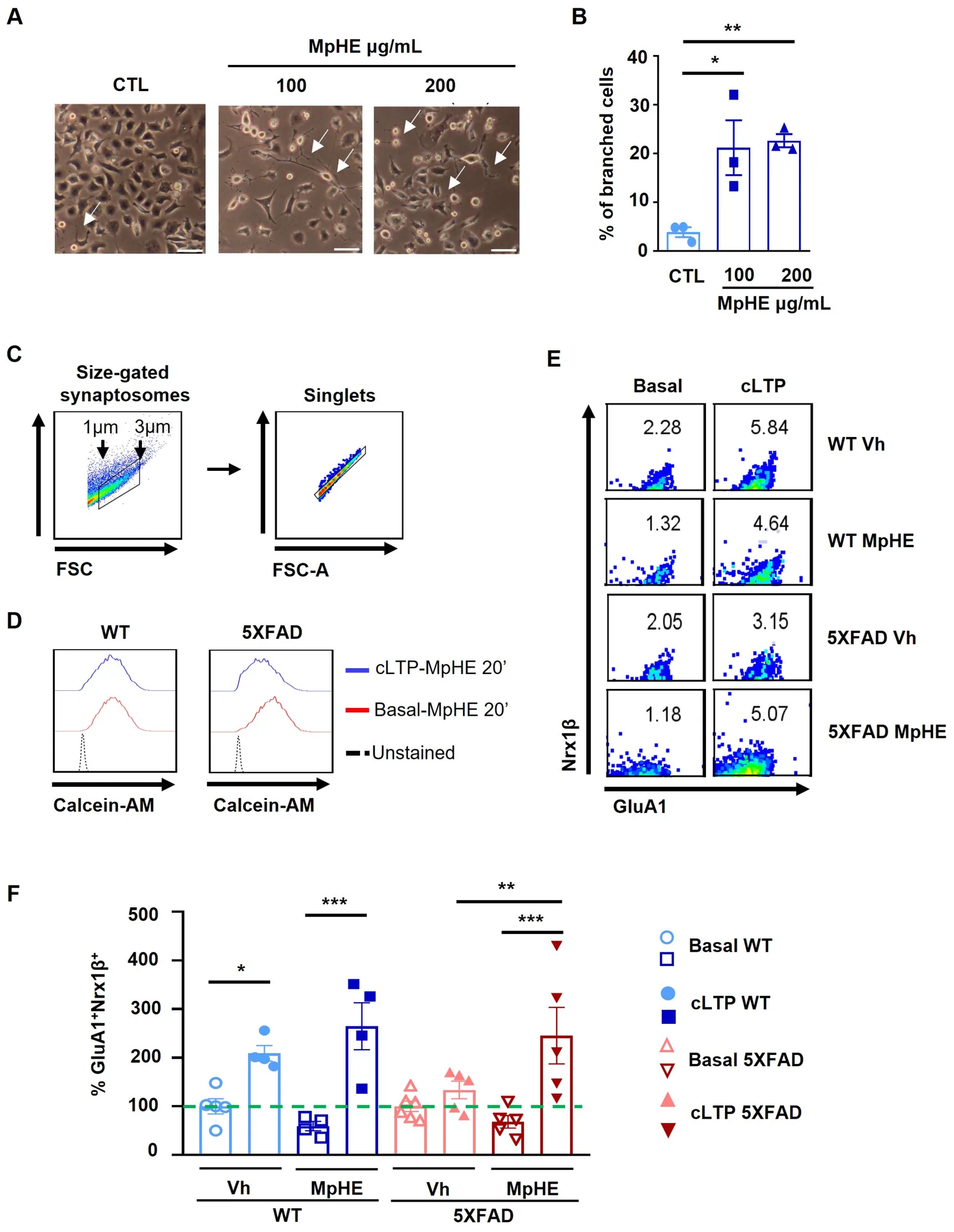
*Malva parviflora* hydroalcoholic extract promotes cholinergic neuronal branching and restores synaptic long-term potentiation. (**A**) Representative microphotographs of SN56 cells treated with MpHE (100 or 200 µg/mL) or control (CTL); arrows indicate branched cells. Images were acquired at 10× magnification using a Nikon Eclipse TS100 optical microscope equipped with a Canon Rebel T7 camera. Scale bar = 30 µm. (**B**) Percentage of branched cells, defined as cells displaying at least one neurite with a length exceeding 1.5 times the diameter of the cell soma. Data represent mean ± SEM; n = 3 independent experiments, 5 fields per condition. (**C**) Size-gated synaptic terminals isolated from WT or 5XFAD female mice, identified relative to calibration beads; singlets were detected using FSC-H vs. FSC-A bivariate plots. (**D**) Calcein-AM viability staining of synaptic terminals from WT (left panel) or 5XFAD (right panel) mice treated with 1 mg/mL MpHE, acquired at the end of the FASS-LTP protocol to confirm synaptosomal integrity. (**E**) Representative flow cytometry plots of the Nrx1β^+^/GluA1^+^ double-positive gate under basal and cLTP conditions in WT and 5XFAD female mice. (**F**) Percentage of size-gated Nrx1β^+^/GluA1^+^ double-positive synaptosomes following cLTP stimulation after pre-incubation with 1 mg/mL MpHE or Vh for 20 min. Values were normalized to the Vh basal condition of each genotype. Statistical significance was assessed by one-way ANOVA followed by the two-stage linear step-up procedure of Benjamini, Krieger, and Yekutieli; n = 4–6 mice per condition; * *p* ≤ 0.05, ** *p* ≤ 0.01, *** *p* ≤ 0.001.

**Figure 4:**
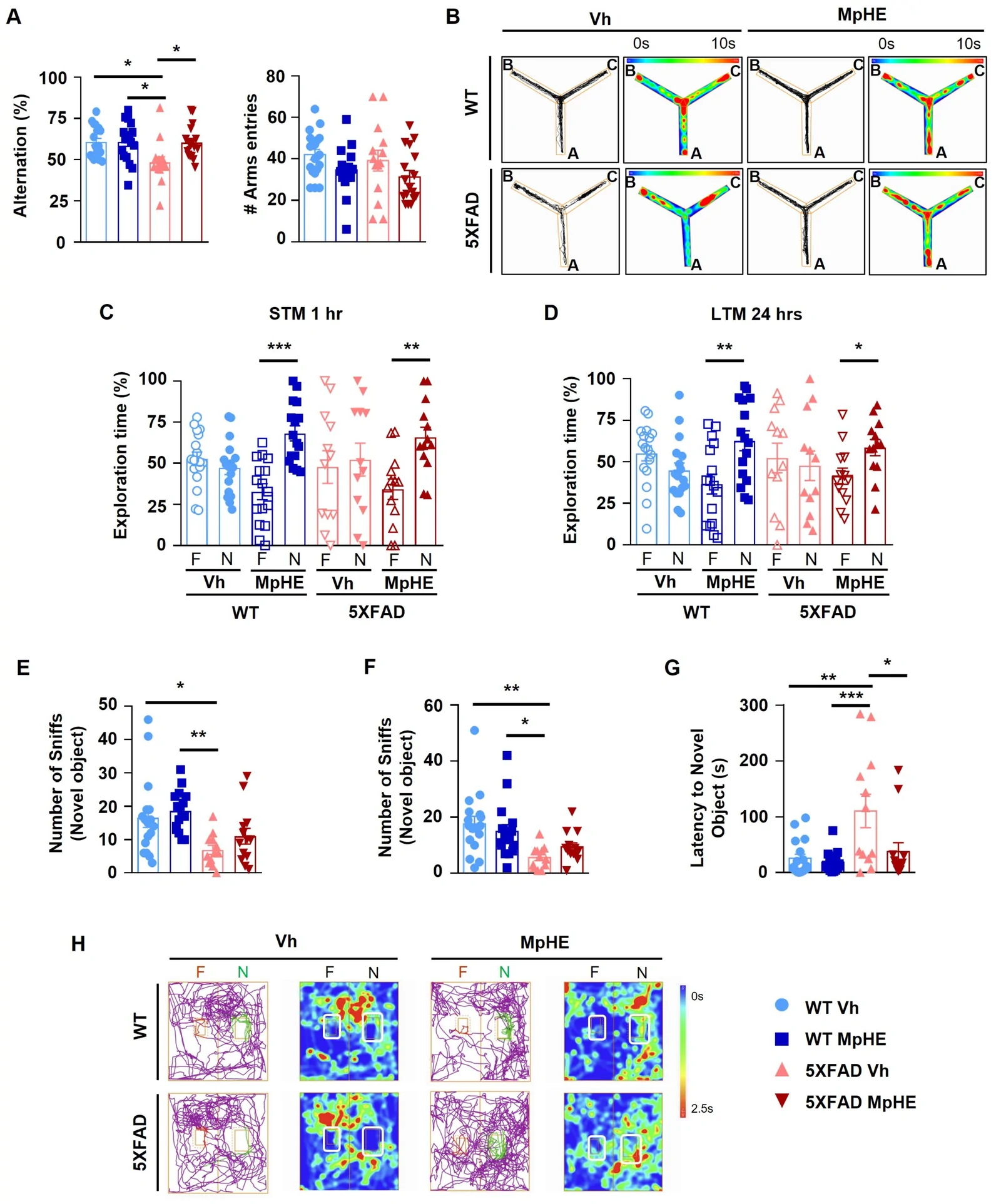
*Malva parviflora* hydroalcoholic extract restores cognitive function in female 5XFAD mice. (**A**) Spontaneous alternation percentage and total number of arm entries in the Y-maze test (WT + Vh: n = 19; WT + MpHE: n = 18; 5XFAD + Vh: n = 14; 5XFAD + MpHE: n = 17). (**B**) Representative track plots and heatmaps of spontaneous alternation performance in the Y-maze. The letters A, B, and C represent the three symmetrical arms of the Y-maze. (**C**,**D**) Novel-object exploration time in the NOR task at 1 h (short-term memory, STM) (**C**) and 24 h (long-term memory, LTM) (**D**) post-training. (**E**,**F**) Number of nose contacts with the novel object at 1 h (**E**) and 24 h (**F**) post-training. (**G**) Latency to first contact with the novel object at 1 h post-training (WT + Vh: n = 17; WT + MpHE: n = 16; 5XFAD + Vh: n = 12; 5XFAD + MpHE: n = 13). (**H**) Representative track plots (purple lines represent the entire path of the mouse) and heatmaps from the NOR task; familiar object (red) and novel object (green). All data are presented as mean ± SEM; n = 12–19 female mice aged 11.5–12 months. Exploration time data were analyzed by unpaired Student’s *t*-test; all other comparisons were assessed by one-way ANOVA followed by Tukey’s multiple comparisons test; * *p* ≤ 0.05, ** *p* ≤ 0.01, *** *p* ≤ 0.001.

## Data Availability

The original contributions presented in this study are included in the article/Supplementary Material. Further inquiries can be directed to the corresponding author.

## Supplementary Materials

The supporting information can be downloaded at https://www.mdpi.com/article/10.3390/ijms27198665/s1.

## Abbreviations

The following abbreviations are used in this manuscript:

Aβ	Amyloid-β
AD	Alzheimer Disease
APP	Amyloid precursor protein
DAA	Disease-associated astrocyte
DG	Dentate gyrus
DS	Dorsal subiculum
GFAP	Glial fibrillary acidic protein
GOT	Glutamic-oxaloacetic transaminase
GPT	Glutamic-pyruvic transaminase
IL-1β	Interleukin 1 beta
IL-18	Interleukin 18
IL-6	Interleukin 6
LTP	Long-term potentiation
cLTP	Chemical long-term potentiation
MpHE	*Malva parviflora* Hydroalcoholic extract
NOR	Novel object recognition
TNF	Tumor Necrosis Factor
UPCR	Urine protein-to-creatinine ratio
Vh	Vehicle
