# Supplementary material for: Therapeutic Administration of Malva parviflora Hydroalcoholic Extract Restores Cognitive Function Consistent with a Resilient Phenotype in Female 5XFAD
Source: Int J Mol Sci. 2026 Sep 28;27(19):8665. doi: 10.3390/ijms27198665 (PMC13664929; doi:10.3390/ijms27198665)
Supplement: Supplementary file 1 [file ijms-27-08665-s001.zip › ijms-4530011-supplementary.pdf]

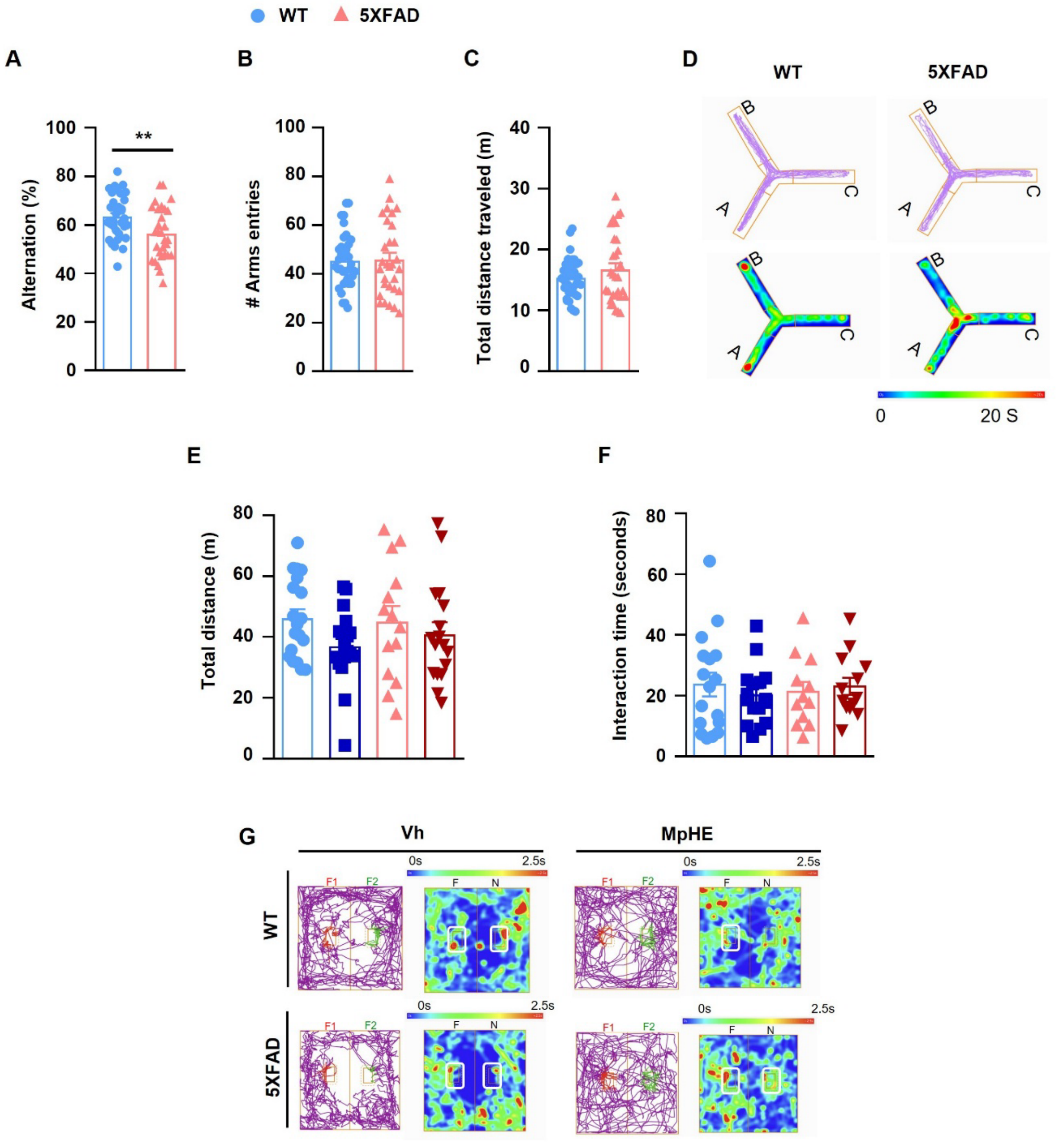

Supplementary figure S1. 5XFAD female mice exhibit impaired working memory before treatment. A) Percentage of spontaneous alternation in the y-maze test. **B**) number of entries in the y-maze test. **C**) Total distance (m) in the y-maze test. **D**) Track plots and heatmaps representative of spontaneous alternation in the y-maze test. **E**) Total distance (m) in the y-maze test after the treatment with MpHE. **F**) Interaction time (seconds) in training phase of NOR. **G**) NOR test track plots and heatmaps. Familiar (F-Red) and novel (N-Green) objects in training phase. **A**-**D**) Before treatment spontaneous alternation, number of arm entries and total distance were analyzed. Data are represented as Mean ± SEM and analyzed by unpaired T-test (**p ≤ 0.0051; n = 30-36 female mice of 6.5 months old. **E-G**) 12–19 female mice of 11.5 months old posttreatment were anaylzed.

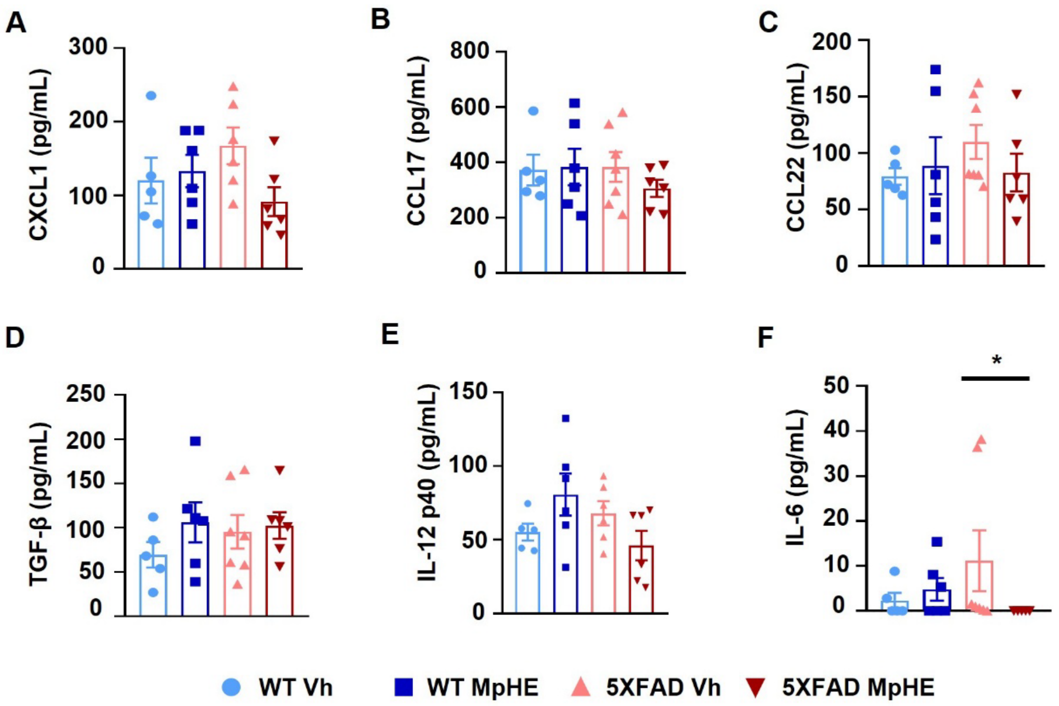

Supplementary figure S2. Serum cytokines levels. CXCL1 (A), CCL17 (B), CCL22 (C), TGF-β (D), IL-12 p40 (E) and IL-6 (F) concentration in the serum of WT and 5XFAD female mice treated with vehicle (Vh) or MpHE was determined using a multi-analyte bead-based flow assay. All data are presented as mean ± SEM. Statistical significance was assessed by one-way ANOVA; *$p \leq 0.05$.

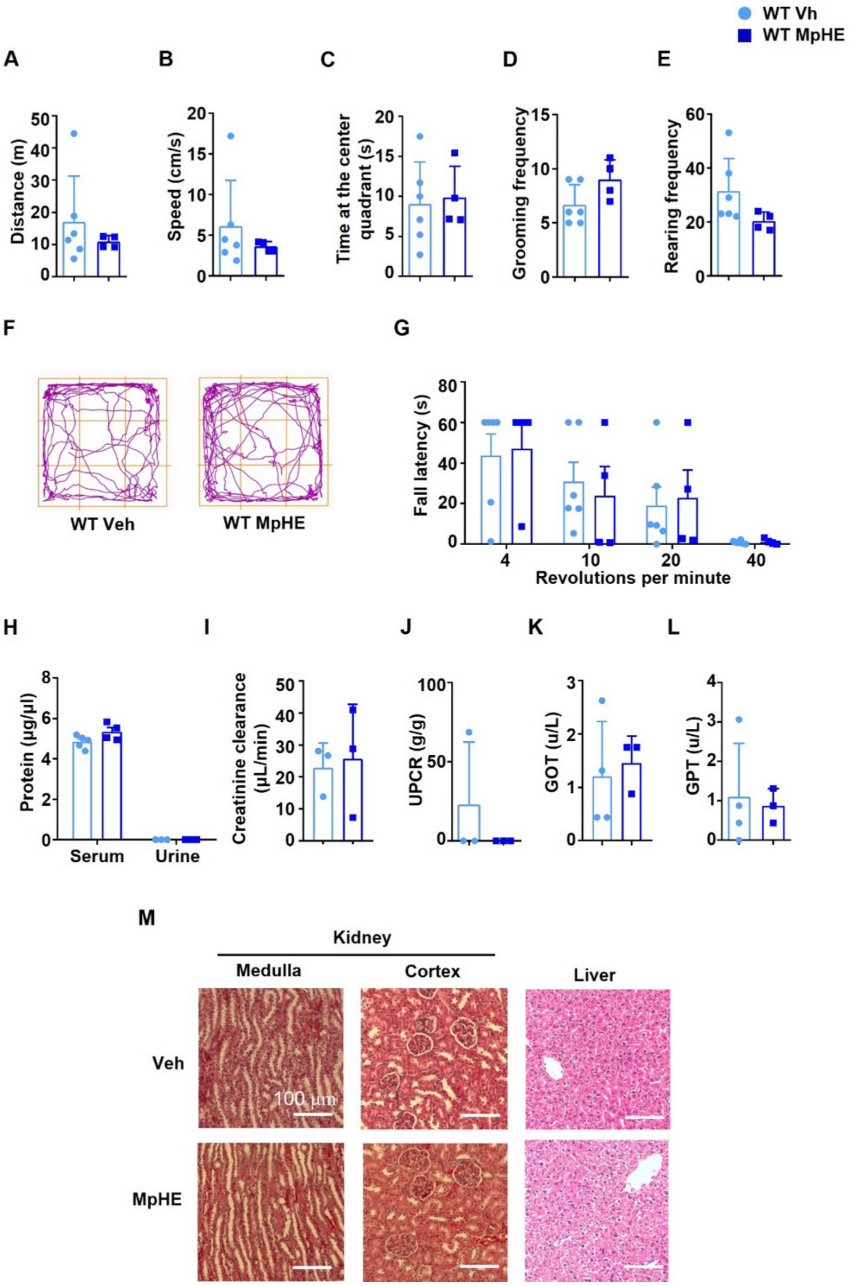

Supplementary figure S3. Malva parviflora´s hydroalcoholic extract safety. MpHE was daily administrated in C57 female mice in a 50 mg/kg dose. Treatment started from approximately the 26th week old and continued for 13 to 15 weeks. Animals were subjected to behavioral tests to determine a possible toxic effect in the central nervous system; and urine, blood, kidney and liver samples were taken to determine organs functionality and tissue integrity. **A**) traversed distance, **B**) average speed, **C**) time spent at the center, **D**) grooming, **E**) rearing frequency and **F**) track plots from the open field test. **G**) Fall latency at 4, 10, 20 and 40 revolutions per minute in the Rotarod test. **H**) Total proteins in serum or urine, **I**) creatinine clearance, and **J**) serum transaminase activity, **K**) glutamic-oxaloacetic transaminase (GOT) and **L**) glutamic-pyruvic transaminase (GPT). **M**) Histological evaluation of kidney and liver tissue by hematoxylin and eosin staining. Data are shown as the mean ± SEM. Statistical analysis was performed by a T-test. (*$p \leq 0.05$; **$p \leq 0.01$; ***$p \leq 0.001$; ****$p \leq 0.0001$). M) tissue integrity observed by hematoxylin- eosin from kidney and liver slices.

Methods Supplementary figure S3:

MpHE was administered daily to C57 female mice at a therapeutic dose of 50 mg/kg. Treatment started at approximately the 26th week old and continued for 13 to 15 weeks. Behavioral, biochemical, and histological tests were performed to determine a general toxicological profile of MpHE. The open field test was carried out using an acrylic platform with a 30 X 30 cm area and clear walls of 15 cm height, the platform base was divided into 9 equal imaginary quadrants and animals were placed at the center quadrant and allowed to freely explore for 5 minutes. The total traversed distance, the average speed, the spent time at the center quadrant, and the grooming and rearing frequency were recorded and quantified. The Rotarod test was performed using a circular rod rotating at a controlled speed. Before the test, mice were placed three times on the rotating rod at 4 revolutions per minute (RPM) to adapt to the instrument. After training, animals were returned to the rotating rod at four increasing speeds (4, 10, 20, 40 rpm), and the fall latency was recorded for each. Twenty-four-hour urine was collected from the animals with the help of metabolic cages, to quantify creatinine-J and total protein. Total protein from serum and urine was determined by the BCA assay kit from Thermo Fisher scientific (CAT 23225) under the producer recommendations. The determination of glutamic-oxaloacetic transaminase (GOT) and glutamic-pyruvic transaminase (GPT), as well as creatinine, from urine or serum, was realized by using the corresponding assay kits from Spinreact (CAT 1001170, CAT 1001160 and CAT 1001111, respectively), following the manufacturer's instructions with some modifications, using a five times smaller final volume to read on a plate-reading spectrophotometer. Kidney and liver were collected, fixed in 4% paraformaldehyde, and embedded in paraffin to obtain one μm thick sections. Sections were then deparaffinized in xylene, rehydrated in graded ethanol series and stained with Hematoxylin and Eosin (H&E).
